# SNPs in predicting clinical efficacy and toxicity of chemotherapy: walking through the quicksand

**DOI:** 10.18632/oncotarget.25256

**Published:** 2018-05-18

**Authors:** Raffaele Palmirotta, Claudia Carella, Erica Silvestris, Mauro Cives, Stefania Luigia Stucci, Marco Tucci, Domenica Lovero, Franco Silvestris

**Affiliations:** ^1^ Department of Biomedical Sciences and Human Oncology, Section of Clinical and Molecular Oncology, University of Bari Aldo Moro, 70124 Bari, Italy

**Keywords:** cancer, targeted therapy, chemotherapy, precision medicine, single nucleotide polymorphisms

## Abstract

In the “precision medicine” era, chemotherapy still remains the backbone for the treatment of many cancers, but no affordable predictors of response to the chemodrugs are available in clinical practice. Single nucleotide polymorphisms (SNPs) are gene sequence variations occurring in more than 1% of the full population, and account for approximately 80% of inter-individual genomic heterogeneity. A number of studies have investigated the predictive role of SNPs of genes enrolled in both pharmacodynamics and pharmacokinetics of chemotherapeutics, but the clinical implementation of related results has been modest so far. Among the examined germline polymorphic variants, several SNPs of *dihydropyrimidine dehydrogenase* (DPYD) and *uridine diphosphate glucuronosyltransferases* (UGT) have shown a robust role as predictors of toxicity following fluoropyrimidine- and/or irinotecan-based treatments respectively, and a few guidelines are mandatory in their detection before therapy initiation. Contrasting results, however, have been reported on the capability of variants of other genes as MTHFR, TYMS, ERCC1, XRCC1, GSTP1, CYP3A4/3A5 and ABCB1, in predicting either therapy efficacy or toxicity in patients undergoing treatment with pyrimidine antimetabolites, platinum derivatives, irinotecan and taxanes. While formal recommendations for routine testing of these SNPs cannot be drawn at this moment, therapeutic decisions may indeed benefit of germline genomic information, when available. Here, we summarize the clinical impact of germline genomic variants on the efficacy and toxicity of major chemodrugs, with the aim to facilitate the therapeutic expectance of clinicians in the odiern quicksand field of complex molecular biology concepts and controversial trial data interpretation.

## INTRODUCTION

The human genome includes 3 billions of nucleotides, and inter-individual sequence variations are detected with a frequency of 1/300-1000 nucleotides. Single nucleotide polymorphisms (SNPs) are germline sequence variations observed in more than 1% of the general population, and account for approximately 80% of the overall genomic heterogeneity [1]. Among the 10 million SNPs identified in the human genome, only 100,000 have a phenotypic and functional impact, since the majority of them is located in intronic portions of the DNA [2, 3].

Functional SNPs are key determinants of inter-individual anthropometric differences, but may also activate the responses to environmental factors and predict the individual disease susceptibility [4, 5]. Moreover, a number of pharmacogenomic studies have demonstrated that both efficacy and toxicity of drugs are largely influenced by SNPs [6], and this event appears particularly relevant in cancer patients receiving chemotherapy since a definite correlation between chemotherapy efficacy/tolerability and survival outcomes, cannot be denied.

The optimization of the so-called “patient-therapy binomial” constitutes one of the main challenges of the modern oncology [7]. In the “precision medicine” era it is imperative, indeed, to match the right patient with the right treatment, and in this context the analysis of clinically meaningful SNPs may provide better efficacy outcomes and, at the same time, decreased treatment-related toxicities. Although a number of studies have investigated the correlation between specific genotypes and response to chemotherapy, clinical implementation of such information has been limited thus far, possibly as consequence of inconclusive results from unrelated studies. Thus, while tumor genotyping is currently routinely used to guide treatment selection in the clinical arena, patient genotyping is considered very often limited to cases of cancer predisposition syndromes, in which the identification of a germline mutation is extremely useful in defining the therapeutic strategy.

In this review, we aimed at updating clinicians with the most recent oncogenomic data deriving from the analysis of selected gene polymorphisms involved in the metabolism of major chemotherapeutic classes including fluoropyrimidines, platinum derivatives, irinotecan and taxanes). In particular, we focused on the biology of the described genetic variants as well as their potential impact as predictors of treatment response or toxicity. Thus, we hope to guide practitioners in: i) requiring the most appropriate molecular investigations; ii) learning the results of genetic reports; and iii) tailoring the therapeutic choices based on the patient genotype, when indicated.

## FLUOROPYRIMIDINES

**Figure 1:**
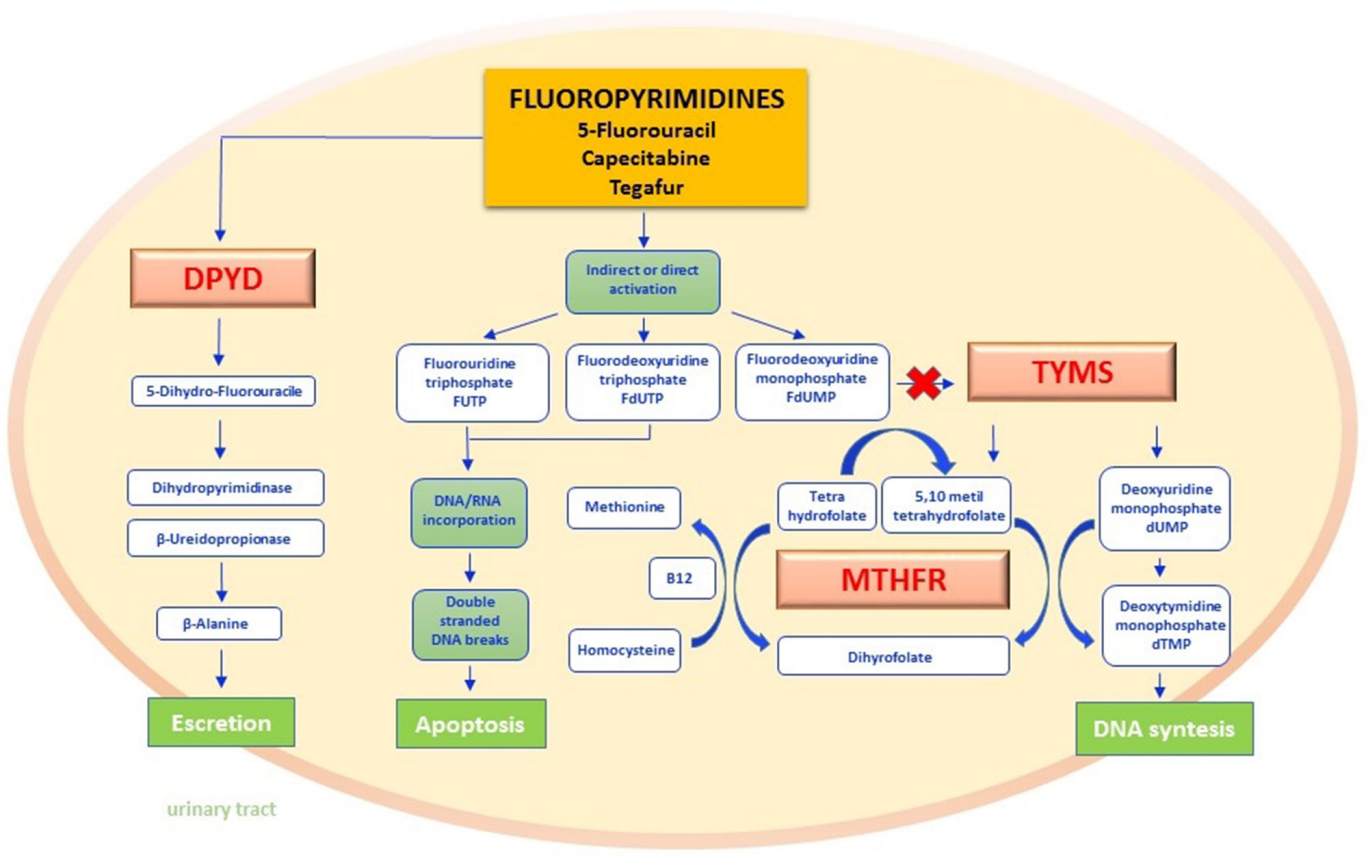
Fluoropyrimidines pathway Fluoropyrimidines (5-fluorouracil and the oral prodrug capecitabine and tegafur) are for the 90% rapidly catabolized in the liver, whereas only 10% is anabolized by forming metabolites responsible for the drug mechanism of action. The rate-limiting step of 5-FU catabolism is catalyzed by dihydropyrimidine dehydrogenase (DPYD) with the synthesis of dihydrofluorouracil (DHFU) and subsequent metabolic reactions lead to the synthesis of inactive compounds excreted by the urinary tract. The main mechanism of action of fluoropyrimidines includes the interaction by either direct or indirect mechanisms, with normal nucleoside biosynthesis. In fact, when active metabolites produced as FUTP, FdUTP, FdUMP are embedded as analogues of pyrimidines in RNA and DNA synthesis, they break the nucleic acid filaments by promoting apoptosis in cancer cells. FdUMP furthermore inhibit the thymidylate synthase (TYMS) enzyme by forming a covalent ternary complex. The inhibition of this reaction not only interrupts the biosynthesis of DNA nucleotides but also interferes with the folate cycle. In this last pathway methylene tetrahydrophilate reductase (MTHFR) is the key enzyme of transmetilation reactions: methyl groups derived from the folate pool in fact permits homocysteine-methionine reconversion by recycling the methyl group bound to Vitamin B12 as a cofactor. DPYD - dihydropyrimidine dehydrogenase; DHFU - dihydrofluorouracil; FUTP - fluorouridine triphosphate; FdUTP - fluorodeoxyuridine triphosphate; FdUMP - fluorouridine monophosphate; TYMS - thymidylate synthase; MTHFR - methylene tetrahydrophilate reductase.

Fluoropyrimidines as fluorouracil, capecitabine and tegafur, have a prominent role in the treatment of many tumors, especially of the gastroenteropancreatic tract, and those of head and neck district [8]. By acting as pyrimidine analogues, fluoropyrimidines cause defects of nucleotide synthesis thereby inducing apoptosis in cancer cells [9, 10]. As depicted in Figure 1, active metabolites of fluoropyrimidines inhibit thymidylate synthetase (TYMS) and inhibit the folate cycle by interfering with the methilentetrahydrofolate reductase (MTHFR) transmetilation reactions [11]. Several enzymes including dihydropyrimidine dehydrogenase (DPYD) concur in the metabolism of fluoropyrimidines [12], and their levels of activity may influence the intracell drug concentration. In the section below, we synthesize both biologic and clinical impacts of DPYD, MTHFR and TYMS polymorphisms.

● **DPYD** - Physiologically, liver DPYD inactivates the 80-90% of administered fluoropyrimidines, converting them in 5-fluoro-5,6-dihydrouracil through a redox reaction exploiting NADPH/NADP^+^ as a cofactor [13, 14]. Two further reactions, catalyzed respectively by dihydropyrimidinase and beta-ureidopropionase, produce the final metabolites that are ultimately excreted with urine [15] (Figure 1). DPYD gene extends for 950 kb on chromosome 1p22 and includes 23 exons [16]. Variations in gene sequence may cause DPYD deficiency, and are transmitted as autosomal recessive inheritance. In affected subjects, the clinical consequences of DPYD deficiency span from absence of signs/symptoms or mere laboratory alterations (increased pyrimidine concentration in blood, urine or liquor) to complex neurological syndro-mes arising at birth or during childhood (seizures, mental retardation, microcephaly, muscle hypertonicity, autism and motor deficits) [17, 18]. Notably, the severity of clinical presentation is directly related to the extent of functional enzyme impairment. In patients receiveing fluoropyrimidine-based chemotherapy, DPYD deficiency may cause a persistent elevation of the blood drug concentration, and is therefore associated with an increased risk of chemotherapy-related toxicities including neutropenia, nausea, vomiting, diarrhea, stomatitis, mucositis, hand-foot syndrome and peripheral neuropathy [19–21].

The individual tolerance to fluoropyrimidine-based chemotherapy is strictly related to specific polymorphic variants of the DPYD gene. In fact, within the about 160 known SNPs affecting this enzyme, approximately 15 of them acquire a clear functional significance [21]. While for some rare variants (^*^3, ^*^7, ^*^8, ^*^9B, ^*^10, ^*^11, ^*^12) the correlation with DPYD's reduced activity is very likely, for others (^*^4, ^*^5, ^*^6, ^*^9A) it still remains unclear [22]. As recently shown in three different metanalysis [23–25], three SNPs of DPYD (^*^2A, ^*^13 and rs67376798) seem to be prioritarily associated with side effects in patients undergoing fluoropyrimidine-based chemotherapy (Table 1).

**Table 1:** Synopses of the major genes variants involved in the metabolism of fluoropyrimidines, platinum derivatives, irinotecan and taxanes

Gene	Polymorfism	Amino acid	Alternative nomenclature	SNP_ID	Ref.
*Fluoropyrimidines*					
DPYD	**IVS14+1G>A**	splice donor variant	DPYD^*^2A	**rs3918290**	OMIM 612779
DPYD	**A2846T**	Asp949Val	-	**rs67376798**	OMIM 612779
DPYD	**T1679G**	Ile560Ser	DPYD^*^13	**rs55886062**	OMIM 612779
MTHFR	**C677T**	Ala222Val	A222V	**rs1801133**	OMIM 607093
MTHFR	**A1298C**	Glu429Ala	E429A	**rs1801131**	OMIM 607093
TYMS	TSER^*^2/TSER^*^3	28 bp repeat in enhancer region	2R/3R	**rs45445694**	OMIM 188350
TYMS	TSER^*^3R G/C	G>C change in the second repeat of the 3R allele	-	rs2853542 rs34743033	OMIM 188350
TYMS	**1494del6b**	I/D of TTAAAG sequence at 1494 position on the 3’-UTR	-	rs151264360 rs869066439	OMIM 188350
*Platinum derivatives*					
ERCC1	**T19007C**	Asn118Asn	-	**rs11615**	OMIM 126380
ERCC1	**C8092A**	3′-untranslated region	^*^197G > T	**rs3212986**	OMIM 126380
XRCC1	**G28152A**	Arg399Gln	-	**rs25487**	OMIM 194360
GSTP1	**A313G**	Ile105Val	GSTP1Val105	**rs1659**	OMIM 134660
*Irinotecan*					
UGT1A	1^*^28	A(TA)6/7TAA	(TA)7/7	**rs34983651**	OMIM 191740
ABCB1	**C3435T**	Ile1145Ile	-	**rs1045642**	OMIM 171050
CYP3A4^*^1B	**-392A>G**	promoter	-	**rs2740574**	OMIM 124010
CYP3A5^*^3	**6986A>G**	splicing defect	-	**rs776746**	OMIM 605325
*Taxanes*					
ABCB1	**C3435T**	Ile1145Ile	-	**rs1045642**	OMIM 171050
ABCB1	**C1236T**	Gly412Gly	-	**rs1128503**	OMIM 191740
CYP3A4^*^1B	**-392A>G**	promoter	-	**rs2740574**	OMIM 124010
CYP3A5^*^3	**6986A>G**	splicing defect	-	**rs776746**	OMIM 605325

The most relevant polymorphisms with the SNP ID and the OMIM reference (PUBMED database) are reported for each gene, in keping with the effects on amino acid substitution, and the possible alternative nomenclatures.

The IVS14+1G>A variant is characterized by a single G>A point mutation in the GT splice donor site IVS14+1, causing the skipping of exon 14 and the consequent synthesis of a truncated, catalytically inactive protein [26, 27]. On the other hand, the 1679T>G variant is characterized by a single aminoacid substitution from isoleucine to serine at codon 560, encoding for a highly conserved, functionally important segment of DPYD [28]. The variant 2846A>T shows a structural alteration that impairs DPYD function by interfering with cofactor binding or electron transport [29]. The three variants are able to decrease DPYD enzyme activity completely or partially, depending on homozygosity or heterozygosity, and the severity of fluoropyrimidine-associated toxicities correlates with the number of functional alleles [30]. Due to the high risk of toxicities, the US Food and Drug Administratin (FDA; http://www.fda.gov/), the Dutch Pharmacogenetis Working Group and the European Medicines Agency (EMA; http://www.ema.europa.eu/ema) do not recommend the administration of fluoropyrimidines to subjects carrying IVS14+1G>A, 1679T>G or 2846A>T variants [21]. On the other hand, both the National Comprehensive Cancer Network (NCCN) and the American Society of Clinical Oncology (ASCO) recommend DPYD pharmacogenetic analysis only in case of suspected toxicities after fluoropyrimidine-based chemotherapy, while the European Society for Medical Oncology (ESMO) suggests the pharmacogenetic test in the pre-therapy setting as an option for selected patients [21, 31–33]. The Clinical Pharmacogenetics Implementation Consortium (CPIC - https://cpicpgx.org/) has recently identified wild type subjects as normal metabolizers and homozygous patients as poor metabolizers, while heterozygous carriers for any combination of the three variants were defined as those having enzyme activity between 30 and 70% compared with the standard [22, 34, 35]. On this basis, CPIC has contraindicated fluoropyrimidine-based therapies in patients with mutated homozygous genotype, while at least a 50% drug dosage reduction was recommended for heterozygous subjects [22, 35]. AIOM (Italian Association of Medical Oncology) and SIF (Italian Society of Pharmacology) suggest the same dose adjustments proposed by the CPIC guidelines in the presence of DPYD variants (Table 2) [36].

**Table 2:** Genotype-phenotype correlations and recommended fluoropyrimidines dose adjustments according to DPYD, MTHFR and TYMS genotypes

Gene (OMIM)	nt./AA variant (rs code)	Transcriptional effects	Functional effects	Clinical effect/reported findings	Dose adjustment	Ref.
**DPYD (#612779)**	IVS14+1G>A c.1905+1G>A DPYD^*^2A (rs3918290)	Heterozygous genotype	DPD activity is reduced of 30-70% than the normal causing persistence of high concentrations of Fluoropyrimidines	Patients can develop toxicity (neutropenia, nausea, vomiting, severe diarrhea, stomatitis, mucositis, hand–foot syndrome and neuropathy)	Administration of 50% of total Fluoropyrimidines dose	[21, 22, 33, 146]
	T1679G I560S DPYD^*^13 (rs55886062)	Homozygous genotype	DPD activity is completely deficient causing persistence of high concentrations of Fluoropyrimidines	Patients develop certainly severe and sometimes life-threatening toxicity (neutropenia, nausea, vomiting, severe diarrhea, stomatitis, mucositis, hand–foot syndrome and neuropathy)	Fluoropyrimidines therapy is contraindicated	
	c.2846A>T Asp949Val (rs67376798)					
**MTHFR (#607093)**	C677T Ala222Val (rs1801133)	T allele leads to lower MTHFR activity	T allele increases Fluoropyrimidines cytotoxicity	C allele is slightly associated with worse outcome while T allele correlates with better response in CRC patients. T allele correlates with gastrointestinal toxicity in CRC patients and with hand-foot syndrome (not with gastrointestinal and hematological toxicities) in CRC capecitabine treated subgroup.	None	[46–49]
	A1298C Glu429Ala (rs1801131)	C allele leads to lower MTHFR activity	C allele increases Fluoropyrimidines citotoxicity	C allele correlates with better response in CRC patients and with hand-foot syndrome but not with gastrointestinal and hematological toxicities in CRC capecitabine treated subgroup.	None	
**TYMS (#188350)**	2R/3R repeat 5’-UTR (rs45445694)	3R allele increases by four times TS mRNA	3R allele reduces Fluoropyrimidines cytotoxicity in cancer cells with lower frequency of side effects in healthy cells	3R allele correlates with Fluoropyrimidines resistance, with a worst outcome with less desease free survival (DFS) and overall survival (OS) In other studies patients carrying the 3R allele did not have a worse outcome 2R / 2R or 2R / 3R genotypes correlate with Fluoropyrimidines sensitivity and better clinical outcome	None	[58–65]
	3R G/C 5’-UTR (rs2853542) rarely 2RC (rs183205964)	C allele correlates with reduced transcriptional activity of TYMS gene	C allele increases Fluoropyrimidines citotoxicity	C allele causes reduced TYMS activity with higher risk of Fluoropyrimidine toxicity	None	
	1494 ins/del 6b (rs16430/rs34489327)	1494 del allele causes TYMS mRNA instability with lower protein expression	1494 del allele increases Fluoropyrimidines cytotoxicity	1494 del allele correlates with greater sensitivity to Fluoropyrimidine-based therapy	None	

● **MTHFR** - The MTHFR gene maps on chromosome 1 (1p36.3) and encodes for a homo-dimeric protein that contributes to the folate metabolism homeostasis as well as to control the turnover of both nucleic acids and aminoacids [37]. As depicted in Figure 1, MTHFR catalyzes the reduction of 5,10 methylene tetrahydrofolate (THF) in 5-methyl-THF, which will serve as methyl group donor in the conversion of homocysteine in methionine [38].

Severe MTHFR deficiency is caused by rare recessive autosomal mutations and is associated with hyperomocysteinemia and hyperomocysteinuria, osteoporosis, growth retard, visual defects, and thrombophilia. Partial enzymatic deficiencies, due to the presence of common polymorphic variants, can generate hyperomocysteinemia especially in the presence of folic acid defect as well as thrombophilia, and increases both prenatal mortality and coronary heart disease risk [39].

The most studied polymorphic variants of MTHFR include C677T and A1298C (Table 1). Both SNPs are characterized by reduced enzymatic activity and their frequency is greater among Caucasians, especially in Italians and Hispanics, and lower among Africans [40]. The 5-fluorouracil, a fluoropyrimidine compound metabolized intracellularly to 5-fluoro-2-deoxyuridine-5-monophosphate (FdUMP) its active form, carries a cytotoxic effect mediating the formation of a ternary complexes between 5-10 methylene THF, TYMS and FdUMP. This complex inhibits the thymidylate and its intracellular levels decreased with consequent suppression of DNA synthesis. Due to the catalytic deficit of the MTHFR, subsequent to its polymorphic variants, the 5-10 methylene THF concentration increased enhancing the formation and stability of the inhibitory complex, thereby the cytotoxic potential of fluoropyrimidines. [41].

The C677T polymorphism causes the substitution of alanine to valine in the aminoacid sequence of exon 4, reducing the catalytic activity of MTHFR while increasing its thermolability. At 37 °C, indeed, the enzymatic activity in subjects with T/T genotype is reduced by approximately 50% with respect to the C/C genotype [42]. However, the increase in the intracellular stocks of folates has the potential to stabilize the three-dimensional structure of MTHFR and improve its enzymatic function [43].

The A1298C SNP is characterized by the substitution of adenine with cytosine and hence of glutamate with alanine in exon 7 and results in a decrease of MTHFR activity [44]. Given the pivotal role of MTHFR in the metabolism of fluoropyrimidines, its polymorphic variants have been investigated as possible predictors of response or toxicity to chemotherapy, but contrasting results have been reported so far. In a metanalysis of 950 patients with advanced colorectal cancer treated with a first-line 5-FU-based therapy, no correlation was found between C667T or A1298C variants and response to therapy [45]. On the other hand, in a metanalysis of 2,402 colorectal cancer patients treated with 5-FU-based chemotherapy who alternatively reported clinical benefit outcomes and/or adverse events, Jennings BA et al. observed a weak association between MTHFR C677T and dismal outcomes [46], whereas a positive correlation between MTHFR SNPs (C677T and A1298C) and response to fluoropyrimidines was shown in 815 Caucasian patients with colo-rectal cancer [47]. In terms of toxicity prediction, a metanalysis of 4,855 colo-rectal cancer patients treated with 5-FU infusion, demonstrated that MTHFR C677T inversely correlates with neutropenia (OR: 0.60; 95% CI: 0.37-0.97) and general toxicity (OR: 0.79; 95% CI: 0.62-1.00) [24]. Similarly, in another study of 450 patients who underwent 4 cycles of fluoropyrimidine-based chemotherapy, both C677T and A1298C variants were not significantly associated with serious hematological or gastrointestinal side effects. However, in the subgroup of patients who received capecitabine, a significant correlation was found between both MTHFR SNPs and hand-foot syndrome (p=0.0046) [48]. In Wang's meta-analysis only in one of three studies applicable for analyzing the association between MTHFR polymorphism and toxicity, the association between the 677T allele and gastrointestinal toxicity was demonstrated (p=0.002) [49, 50]. In a recent study conducted in two cohorts of stage II/III of CRC patients treated with adjuvant fluoropyrimidine chemotherapy, the MTHFR 1298CC genotype carriers underwent worsened disease free survival and overall survival in both cohorts [51]. Similarly, an even more recent study of 242 Korean patients with mCRC showed that the presence of a C677CC genotype was associated with a good prognosis in multivariate analysis [52]. On the basis also of the significant ethnic differences in the C677T and A1298C genotypes frequency, larger studies including different populations are needed to determine the role of these polymorphisms in response to fluoropyrimidines [53]. Therefore, despite the result of such heterogeneous findings, no formal indications are suggested regarding the clinical genotyping of MTHFR in patients candidates to fluoropyrimidine-based treatments although it is likely that the presence of these polymorphisms in homozygosity must be taken into account during the therapy planning. (Table 2) [54, 55].

**Figure 2:**
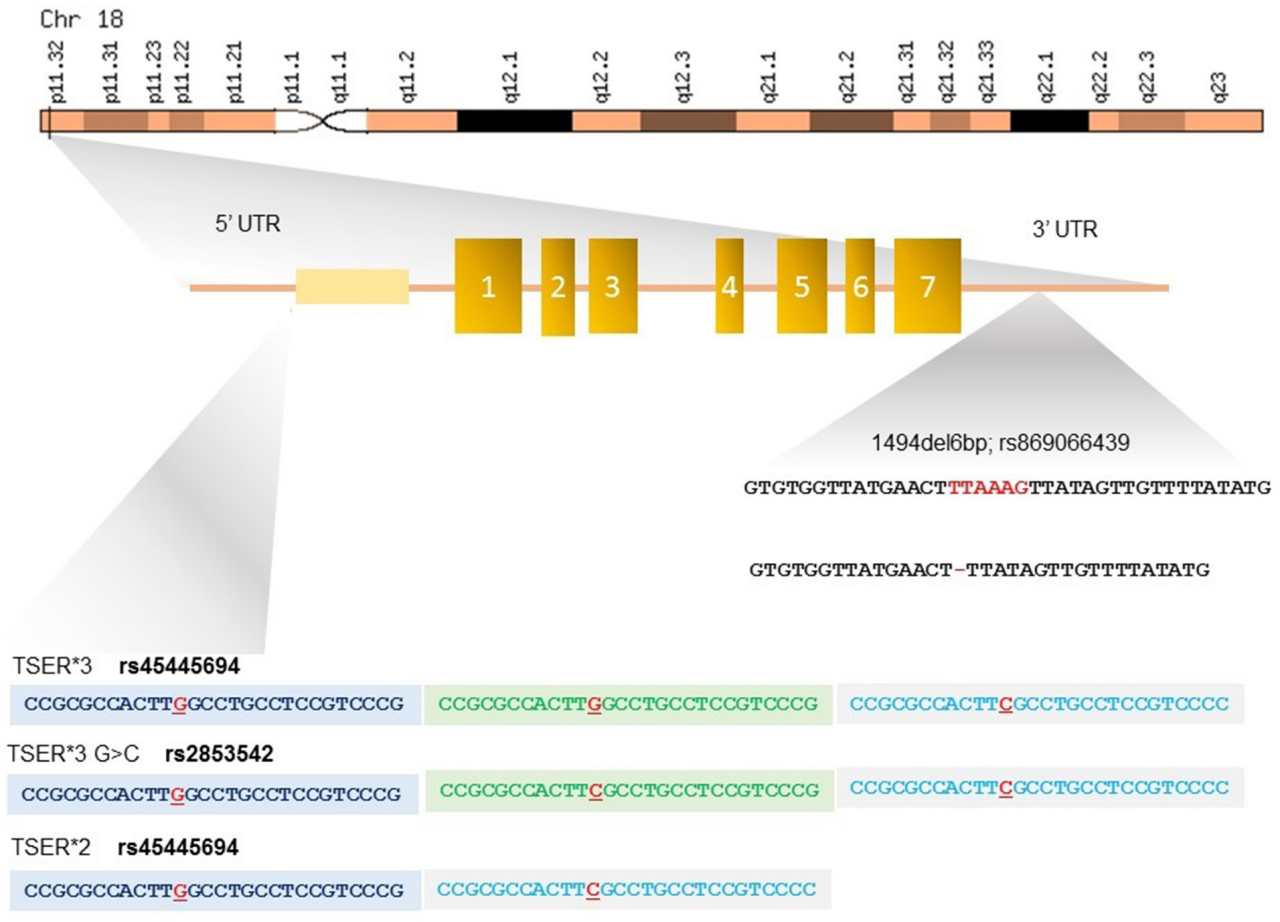
Polymorphisms in the 5’- and 3’-untranslated regions (UTRs) of TYMS gene The 5’-UTR of TYMS, named thymidylate synthase enhancer region (TSER), contains a variable number of a 28-bp double (2R) or triple repeats (3R) determining the genotypes 2R/2R, 2R/3R and 3R/3R. TYMS expression level is directly proportional to the number of repeats. The TSER^*^3R G/C polymorphism consists in a G>C change in the second repeat of the 3R allele and results in a lower transcriptional activation of TYMS. The 3’-UTR insertion/deletion of hexanucleotide TTAAAG in position 1494 is in linkage disequilibrium with the TSER 3R allele. The -6 bp deletion results in a 70% decrease in TYMS mRNA levels.

● **TYMS** - TYMS is located in the short arm of chromosome 18 (18p11.32), contains 7 exons and spans about 30 kb. TYMS is a folate-dependent enzyme and competes with MTHFR for the availability of the cofactor 5,10 methylene THF that catalyzes the reductive methylation of deoxyuridylate (dUMP) to thymidylate (dTMP), thereby playing a central role in DNA synthesis and repair [56] (Figure 2). 5-FU efficacy is directly correlated with TYMS expression levels [57] and one of the major determinants of TYMS expression is the presence of three polymorphic variants, namely TSER^*^2R/^*^3R (rs45445694), TSER^*^3R G/C (rs2853542 –rs34743033) and 1494del6b (rs151264360 – rs869066439) [58] (Table 2).

The 5’-UTR of TYMS contains a variable number (from 2 to 9) of a 28-bp tandem repeat sequence (VNTR) that acts as enhancer for the promoter by implementing the gene transcriptional activity (Figure 2). Polymorphic variants of this region, named thymidylate synthase enhancer region (TSER), have been described, and include double (2R) or triple repeats (3R) determining the genotypes 2R/2R, 2R/3R and 3R/3R [59]. Functional studies have shown a stepwise increase in TYMS transcription with an increasing number of tandem repeats [60], and patients with metastatic colo-rectal cancer with a 3R/3R genotype show 3.6 times higher mRNA levels as compared with those homozygous for 2R/2R [61]. The higher TYMS expression level related to the presence of 3R/3R genotype accounts for less effective inhibition of TYMS and worse response to fluoropyrimidines, in presence of low toxicity. Conversely, the occurrence of two tandem repeat polymorphism (2R/2R or 2R/3R genotype) leads to more favorable responses to 5-FU treatment, [62, 63] but may enhance the 5-FU toxicity (Table 2) [58].

The TSER^*^3R G/C polymorphism consists in a G>C change in the second repeat of the 3R allele (rs2853542 – rs24743033) (Figure 2). This SNP is associated with a weaker bond in the promoter region of upstream transcription factor 1 (USF1) and results in a lower transcriptional activation of TYMS. It is found in 30%-55% of all TSER 3R alleles and its presence explains why not all patients with the TYMS 3R allele have poor outcomes (Table 2) [64, 65].

Besides these variants, an insertion/deletion of hexanucleotide TTAAAG sequence at 1494 position on the 3’-UTR of the TYMS gene, has been also described to be in linkage disequilibrium with the TSER 3R allele and is associated with worse prognosis in 5-FU treated patients [66]. The -6 bp deletion results in a 70% decrease in mRNA levels [67], probably as effect of the accelerated degradation of the transcript. Thus, by combining the above mentioned SNPs, it is possible to predict high levels of TYMS expression in subjects with 2R/3RG, 3RC/3RG, 3RG/3RG; +6bp/+6bp genotypes, and conversely low levels of the enzyme in those with 2R/2R, 2R/3RC, 3RC/3RC; -6bp/-6bp, -+6bp/-6bp genotypes [58].

Although knockdown or amplification experiments succeeded in demonstrating the *in vitro* importance of TYMS in 5-FU resistance and toxicity, clinical studies on the predictive role of these polymorphisms have been controversial so far [68]. For example, no significant association between TYMS genotype and response rate or overall survival (OS) has been observed in patients with gastric cancer treated with platinum/5-FU combinations. The same study found individuals with a 3R haplotype to have a significantly lower risk of developing grade 3/4 leukopenia after chemotherapy [49]. In the mentioned metanalysis performed by Jennings BA et al., on 2,402 patients with colo-rectal cancer, the 2R/2R genotype was apparently characterized by a significantly higher risk of toxicities when compared with the 2R/3R and 3R/3R genotypes [46], whereas the toxicity to capecitabine appeared globally greater in carriers of 2R/3R and 6bp insertion polymorphisms [24]. However, no association between capecitabine efficacy and TYMS polymorphic variants has been reported so far [64, 69] and, as for MTHFR, no specific indications have been formulated thus far for the clinical testing of TYMS SNPs in cancer patients who may benefit of fluoropyrimidine treatment.

## PLATINUM DERIVATIVES

Platinum derivatives are highly efficacious against a broad spectrum of solid tumors and currently constitute the backbone for the treatment of pulmonary, head and neck, gastroentero-pancreatic and genitourinary neoplasms [70].

**Figure 3:**
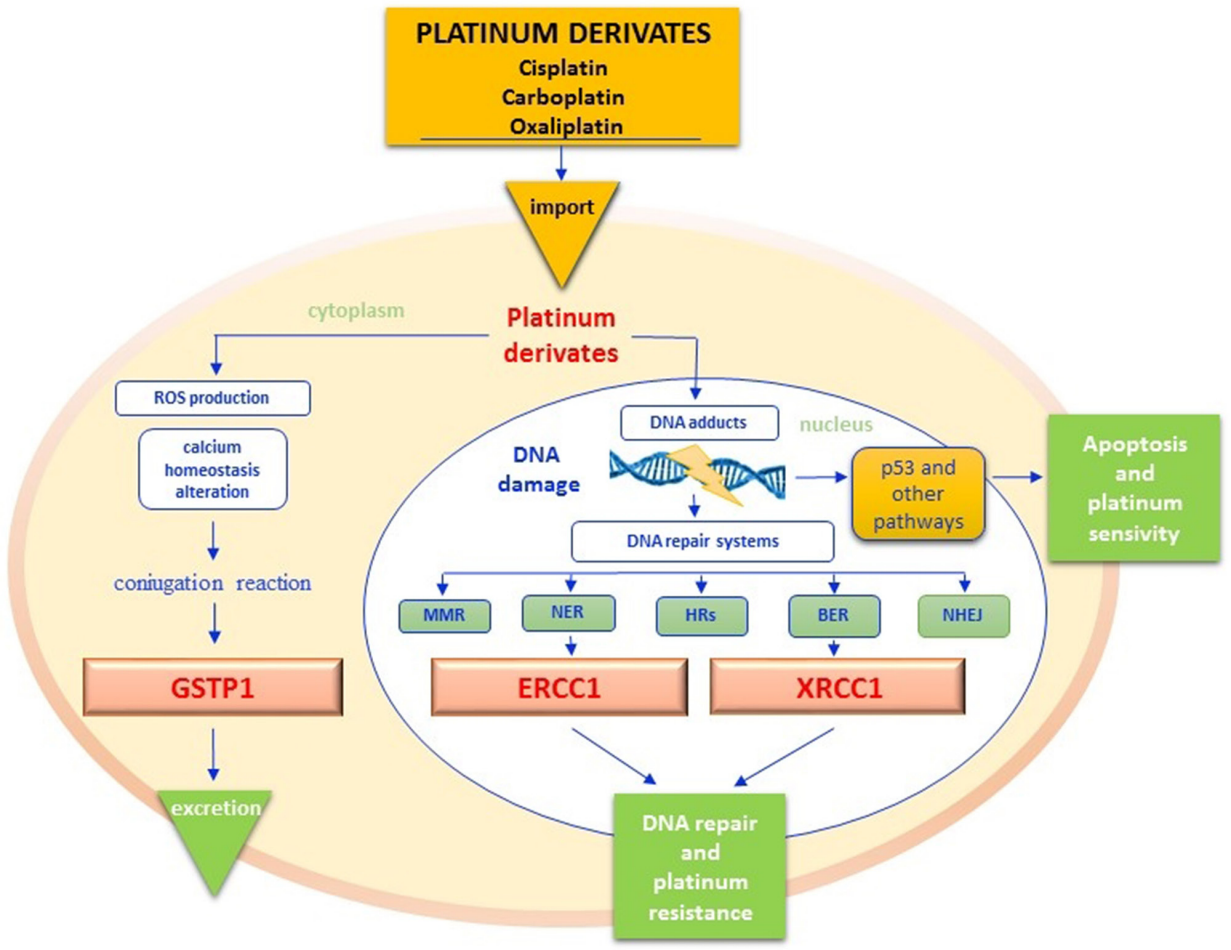
Platinum pathway Once into cytoplasm, platinum derivatives promote the Reactive Oxygen Species (ROS) synthesis, that cause the alteration of cell membranes permeability, the deregulation of different signal transduction pathways and calcium homeostasis but overall the DNA damage. Glutathione S-transferases protein 1 (GSTP1) catalyzes the conjugation reaction of platinum derivates with reduced glutathione (GSH), in order to increase their hydro-solubility and to facilitate their excretion. When platinum derivatives reach the nucleus, they form intra and interstrand DNA cross-links that block the cell cycle by activating tumor cell apoptosis through different pathways. DNA adducts however may activate sensor proteins and DNA repair systems by avoiding cytotoxicity. Excision repair cross complementation group 1 (ERCC1) is the main endonuclease of DNA NER (Nucleotide Excision Repair) pathway but it also interacts with the BER (Base Excision Repair) function in maintaining chromosomal stability and telomers integrity. X-ray repair cross-complementing group 1 (XRCC1) is another enzyme of BER pathway that repairs DNA bases damaged by X-rays, ROS and mostly alkylating agents (. The efficiency of the GSTP1detoxification reaction and of DNA repairing systems affects the platinum-based treatments response. ROS - reactive oxygen species; GSTP1 - glutathione s-transferases protein 1; ERCC1 - excision repair cross complementation group 1; NER - nucleotide excision repair; BER - base excision repair; XRCC1 - X-ray repair cross-complementing group 1.

Resistance to platinum salts is mainly caused by the hyperactivation of DNA repair systems, with consequent decrease of pro-apoptogenic DNA adducts. As depicted in Figure 3, different enzyme groups are enrolled in DNA repair, including those of the nucleotide excision repair (NER) system, base excision repair (BER) system and mismatch repair (MMR) system [71]. The most representative enzymes of the NER and BER systems include the excision repair cross complementation group 1 (ERCC1) and X-ray repair cross-complementing group 1 (XRCC1) respectively. In addition, homologous (HRs) and non-homologous recombination systems end joining (NHHRsEJ) are involved in DNA repairing processes [71] (Figure 3). Cisplatin is characterized by a strong emetic effect and has a remarkable toxicity profile for kidney, liver, heart and auditory apparatus, as well as severe myelo- and neuro-toxicity [72]. Detoxification of platinum derivatives involves the conjugation with reduced glutathione (GSH), a reaction catalyzed by glutathione S-transferase protein 1 (GSTP1) [73]. Given the key role of enzymes involved in either DNA repair or drug metabolism in the pharmacodynamics and pharmacokinetics of platinum salts, several variants of both ERCC1, XRCC1 and GSTP1 have been investigated as potential biomarkers of either response or toxicity (Table 1).

● **ERCC1** - ERCC1 is a 297 amino acid protein encoded by a gene localized on 19q13 chromosome. After heterodimerization with XP-F, ERCC1 contributes to elimination of DNA adducts induced by UV light, ROS, environmental mutagens and especially by cancer chemodrugs [74] (Figure 3). Moreover, the protein has a role in the preservation of chromosomal stability and telomers’ integrity [75]. High levels of ERCC1 have been associated with platinum resistance, while ERCC1-defective cells appear to be highly sensitive to alkylating agents [76, 77]. The best carachterized SNPs of ERCC1 include the T19007C (Asn118Asn; rs11615) and the C8092A (rs321298) variants [78] (Table 1).

The synonym variant T19007C, although not causing any aminoacid change, results in the low-use codon AAT, instead of the high-use codon AAC, thus significantly reducing the efficacy of ERCC1 mRNA [79]. On the other hand, a reduced expression of ERCC1, as result of the C-allele, has been shown to correlate with better responses to platinum-based therapies in non small cell lung cancer (NSCLC) patients, whereas the T-allele was found to correlate with platinum-resistance in gastric, ovarian and cervical cancers [80–83] (Table 3). Several retrospective studies have also demonstrated that NSCLC patients carrying the T-allele have poor overall survival when subjected to platinum-based chemotherapy [84, 85] and the association between the T-allele presence and dismal outcomes has been confirmed in patients with metastatic colo-rectal cancer treated with platinum. In fact, the median overall survival (OS) was as low as 15.3 months in subjects with C/C genotype, and 11.1 months in C/T or T/T carriers [86, 87].

**Table 3:** ERCC1, XRCC1 and GSTP1 genes variants, potential impact on the enzyme activity and outcome of platinum derivatives therapies

Gene (OMIM)	nt./AA variant (rs code)	Transcriptional effects	Functional effects	Clinical effect/reported findings	Dose adjustment	Ref.
**ERCC1 (#126380)**	T19007C Asn118Asn (rs 11615)	C allele leads to lower ERCC1 expression	C allele increases platinum genotoxicity	C allele correlates with higher response rate in NSCLC patients, also in adjuvant setting and Asian patients. C allele predicts FOLFOX response, better OS and PFS in mCRC patients. T allele correlates with platinum resistance in gastric, ovarian, cervical and other cancers and with a reduced OS in NSCLC patients. T allele is a biomarker of low objective FOLFOX response, lower PFS and OS in Asian gastro-intestinal tumor patients but not in Caucasians. T allele is also independently associated with worse PFS in mCRC patients and with high risk of local recurrence and worse clinical outcome in association with ERCC2 codon751 A/A variant.	None	[79–92]
	C8092A (rs321298)	A allele affects mRNA stability causing a lower enzyme expression	A allele increases platinum genotoxicity	A allele is associated with an increased risk of grade 3 or 4 gastrointestinal toxicity and with anemia in advanced NSCLC patients in mCRC patients. The combination of T118 allele and A8092 allele correlate with worse ORR and OS compared with the C allele in the Asian NSCLC sub-group patients, but not in Caucasians ones.	None	[95–97]
**XRCC1 (#194360)**	G28152A Arg399Gln-(rs25487)	A allele leads to lower XRCC1 expression	A allele increases platinum toxicity and tumor aggressiveness	A allele correlates with grade 3- 4 gastrointestinal and hematologic toxicities in NSCLC patients. A allele is associated with worse ORR, OS and clinical outcome in NSCLC, gastric and CRC patients Conversely another study correlated A allele with better OS in other CRC patients.	None	[102, 107, 108, 110–116]
**GSTP1 (#134660)**	A313G Ile105Val (rs1659)	G allele leads to lower GSTP1 activity	G allele increases platinum genotoxicity	G allele correlates with grade 3 neurotoxicity in mCRC patients while in NSCLC patients G allele has none toxicities associations. G allele is associated with better outcome and OS in breast, CRC, NSCLC and gastric cancer patients but Another study didn't show associations with PFS in CRC patients	None	[109, 113, 123–132]

Other studies observed a different genotype-phenotype correlation between ethnic subgroups, by showing that the 1907T allele was associated with unfavorable PFS and OS in Asian, and alternatively, with favorable prognosis in Caucasians, probably due to the complexity of interactions between genes and environment, rather than to polymorphism frequencies in each group [54, 88].

Also, within the mCRC patients population, another study on 168 Chinese patients treated with first-line FOLFOX-4 chemotherapy showed that the CC genotype was associated with a better response rate and clinical outcome (ORR: 57.5% vs 36.4%; p = 0.01), as well as PFS (13 months vs 7 months; P < 0.01), and OS (25 months vs 16 months; P < 0.01) compared to CT ot TT genotypes [89].

Other studies included a metanalysis conducted on 1,787 gastric and colon cancer patients treated with oxaliplatin-based chemotherapy demonstrated the role of rs11615 T allele as a predictor of low objective response, shorter PFS and OS in Asian, but not Caucasian people [90]. The same association was shown in a subgroup analysis of another metanalysis, reflecting the strong influence of ethnicity-dependent factors in pharmacogenetic assay [88]. The combination of several enzymatic variants involved in fluoropyrimidines and platinum metabolism has been also reported to correlate with PFS after first-line chemotherapy in patients with metastatic colo-rectal cancer. In particular, the combination of ERCC1-118 T/T, ERCC2-751 A/C, and ERCC2-751 C/C was independently associated with low PFS in 166 patients [91] since in post-operative colorectal cancer evolution of 257 Taiwanese patients, the ERCC2-751 A/A and ERCC1-118 T/T genotypes predicted higher incidence of recurrence and worse clinical outcome [92].

Another common variant of ERCC1 is C8092A. This SNP is located in the 3’UTR of the gene and can alter polyadenylation, translation efficiency, localization and stability of mRNA [93]. In particular, the presence of allele A reduces the stability of the ERCC1 transcript, thus resulting in lower protein expression and increased sensitivity to genotoxic chemotherapies [94]. In a recent metanalysis of 33 studies involving nearly 5,000 patients with NSCLC treated with platinum-based chemotherapy, the TT/TC genotypes of the C118T variant and the AA/CA genotypes of the C8092A SNP were associated with lower objective response rate (ORR) and OS as compared with CC genotype. However, this effect was observed only in the Asian population, but not in Caucasian patients [95].

The role of ERCC1 variants as predictors of toxicity following platinum therapy has been poorly investigated. In patients with advanced NSCLC, the 8092A allele, but not the 118T allele, appeared associated with a significantly increased risk of gastrointestinal grade 3 or 4 toxicity [96]. In another study of patients with colorectal cancer treated with platinum-based adjuvant chemotherapy, the 8092A allele was reported to predict hematologic toxicity, in particular anemia [97] (Table 3).

● **XRCC1** - The human XRCC1 protein is encoded by a gene mapping on 19q13.2 chromosome and plays a pivotal role in the BER pathway, replacing DNA bases damaged by X-rays, reactive oxygen radicals and alkylating agents [98–100] (Figure 3). More than 300 SNPs have been shown to affect XRCC1, but only three of them have been functionally characterized. In fact, Arg194Trp, Arg280His and Arg399Gln cause amino acid substitutions in the XRCC1 protein resulting in the alteration of its function [101]. Such polymorphisms have been associated to a general increased cancer risk in the full population as result of impaired capacity of DNA repair, correlation with greater tumor aggressiveness, and lower response to platinum derivatives [102, 103].

The G28152A variant, also named Arg399Gln or rs25487, is the most well-studied SNP of XRCC1 and maps on the COOH-terminal domain of the gene, coding for a protein portion devoted to protein-protein interactions (Table 1) [104, 105]. The 28152A allele is responsible of a substantial defect of XRCC1 to repair DNA, in particular after exposure to ionizing radiation [98, 106].

In NSCLC patients, the A/A or G/A genotypes have been associated with increased risk of all toxicities as compared with the G/G genotype, and particularly with a 2.5-fold increased risk of grade 3 or 4 gastrointestinal toxicities [107]. Also, higher incidence of severe hematologic adverse effects has been observed in carriers of the A allele in another study on 487 NSCLC patients treated with cisplatin, docetaxel and gemcitabine [108]. However, no significant association between XRCC1 399A and chemotherapy-induced toxicities was found in the TOSCA trial, which evaluated 3,579 patients with colorectal cancer treated with FOLFOX-4 or XELOX adjuvant chemotherapy (Table 3) [109].

The G28152A variant has been also investigated as biomarker of response and survival after platinum-based chemotherapy. In a study of 112 NSCLC patients, a progressive increase in average survival times following platinum treatment has been identified in carriers of the A/A, A/G and G/G genotypes respectively [102]. By contrast, a metanalysis of 22 studies investigating platinum-based chemotherapy in advanced NSCLC demonstrated the role of A/A + G/A genotypes in predicting objective responses [110], while worse outcomes were reported for carriers of the A allele in another study of 235 patients with NSCLC treated with platinum [108].

Better outcomes following FOLFOX therapy have been consistently observed in patients with metastatic colo-rectal cancer carrying a G allele [111, 112]. More recently, the presence of the A allele has been instead associated with lower tumor response after oxaliplatin-based chemotherapy in a cohort of 1,234 patients with colorectal cancer. Surprisingly, no correlation with PFS was found [113]. In gastric cancer patients treated with oxaliplatin-based chemotherapy, the A allele conferred a significant disadvantage in terms of survival [114]. Contradictory evidence has been generated on the prognostic role of XRCC1 399A in patients with colo-rectal cancer [115, 116].

● **GSTP1** - The glutathione S-transferases (GSTs), subdivided in seven enzymatic classes (α, μ, κ, τ, π, ω and ζ) detoxify mammalian cells by endogenous and exogenous, hydrophobic and electrophilic toxic compounds by using reduced glutathione (GSH), thus avoiding the formation of DNA adducts (Figure 3). The gene of Pi-class glutathione-S-transferase (GSTP1) maps on chromosome 11q13.2, extending for about 2.8 Kb [117]. GSTP1 catalyzes the conjugation of platinum derivatives with reduced glutathione (GSH), in order to increase their hydro-solubility and excretion [73]. Several *in vitro* studies have shown a significant correlation between resistance to platinum and high levels of intracellular GSH, as well as between platinum resistance and elevated GSTP1 expression [118–120].

Seminal studies on the functional polymorphisms of the GSTP1 identified two variants of the gene (A313G and C114T), whose combinations result in four functional haplotypes GSTP1^*^A (AC); GSTP1^*^B (GC), GSTP1^*^C (GT) and GSTP1^*^D (AT) [121]. In this context, the G/G genotype at nucleotide 313 (A313G; Ile105Val; rs1659)seems to substantially decrease the enzymatic activity of GSTP1, as it causes an aminoacid substitution in the active site of the protein [122]. As consequence, a possible implication of GSTP1 G-harboring SNPs in the response to platinum derivatives has been envisaged [73] GSTP1 genotypes G/G or G/A with lower enzyme activity potentially correlate with increased response to platinum-based chemotherapy due to the decreased detoxification activity [73]. The G/G genotype of GSTP1 has been associated with grade 3 neurotoxicity in patients with colo-rectal cancer who received FOLFOX [91] (Table 3). This observation was also confirmed in a study of n166 Asian patients with metastatic colorectal cancer, and similar findings were reported for Caucasion patients with inoperable NSCLC treated with platinum-gemcitabine [123, 124]. However, not all studies have been consistent in demonstrating a significant association between platinum salts, neurotoxicity and GSTP1 variants enriched in alleles G [125, 126].

While causing increased toxicities, the reduced detoxifying activity of GSTP1 determined by the G/G genotype has been also hypothesized to lead to better clinical outcomes in response to platinum-based protocols. However, contrasting data have been reported at this regard in several cancers [91, 127, 128], and a large metanalysis of 13 studies on colo-rectal cancer patients failed to show any association between the G allele and PFS [113]. Ethnic differences among enrolled patients (46% Caucasian, 64% Asian) may have probably impaired the analysis [54]. Even in NSCLC patients, different metanalyses and clinical studies have shown the association between G/G genotype and increased platinum-based chemotherapy efficacy in terms of both response rates and OS [129–131]. The positive predictive role in terms of tumor response, PFS and OS of the G/G genotype was also confirmed in a recent metanalysis including 8169 cases with gastric cancer subjected to platinum-based chemotherapy [132].

## IRINOTECAN

**Figure 4:**
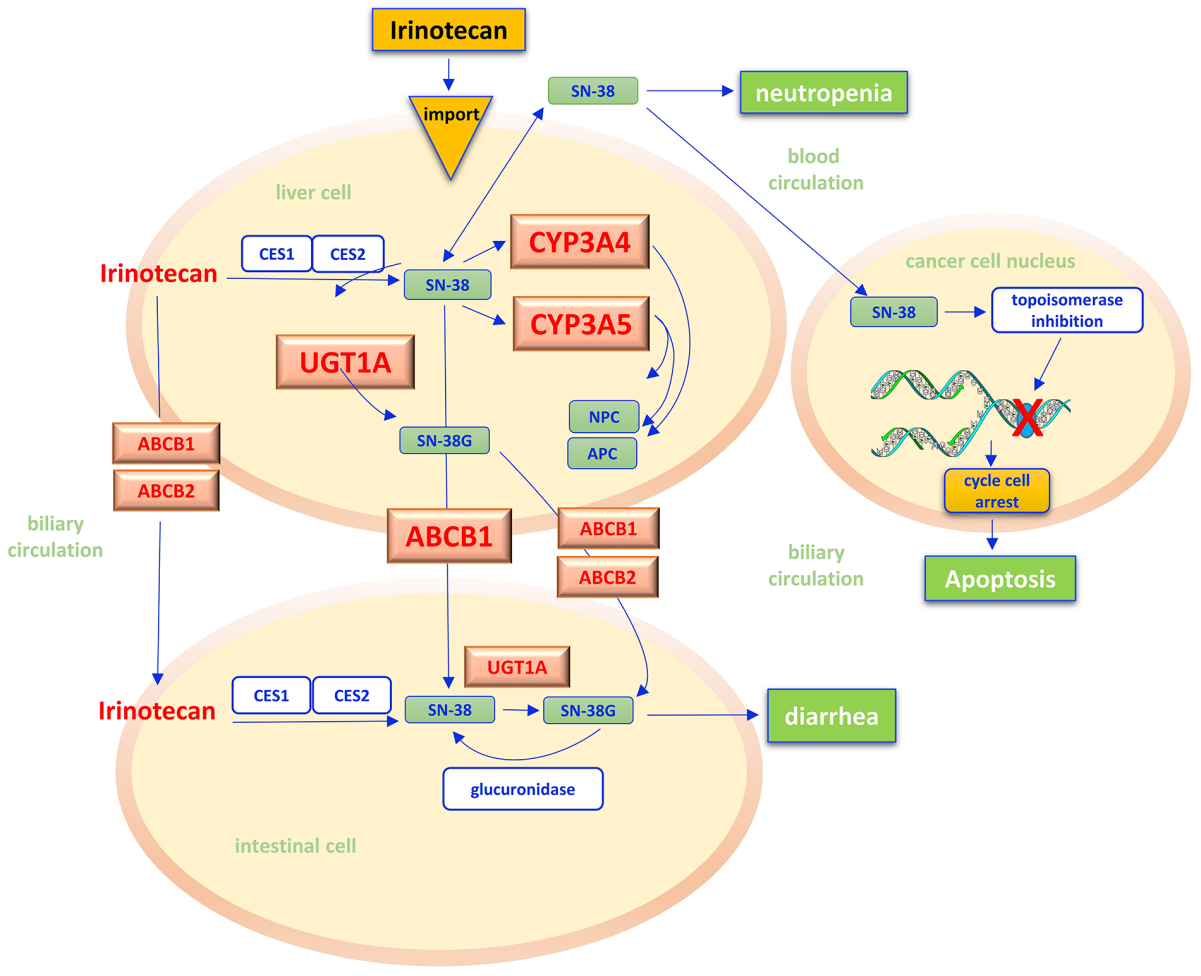
Irinotecan pathway Irinotecan is a prodrug that, after administration, is activated in liver by the hydrolysis reaction catalyzed by carboxylesterases (CES1, CES2) of the microsomal system of hepatocytes, with the release of the more active metabolite SN-38. In cancer cell nuclei, SN-38 acts as inhibitor of the topoisomerase I, a key enzyme in DNA replication. The SN-38/enzyme/DNA complex causes major breaks in the DNA replication fork, with subsequent activation of cancer cells apoptosis. In liver cells, irinotecan and SN-38 may be oxidated by hepatic cytochrome P-450 (CYP 3A4 and 3A5) to form pharmacologically inactive metabolites (NPC, APC). The Uridine diphosphate (UDP) glucuronosyltransferases (UGT) catalyzes the subsequent conjugation reaction of SN-38 with glucuronic acid making its excretion possible through the bile in the intestinal lumen. Adenosine-triphosphate binding cassettes (ABC) transporters (ABCB1/ABCB2) are transmembrane proteins which make possible the absorption of SN-38 from plasma into hepatocytes and hence in interstitial and the excretion of irinotecan and its metabolites by bile into the intestinal lumen. An increased bioavailability of SN-38, i.e. for the reduced efficiency of UGT and CYP 3A4/3A5 reactions, seems to justify the onset of diarrhea and neutropenia as specific side effects of chemotherapy. CES – carboxylesterases; CYP - cytochrome P-450; UGT - uridine diphosphate (UDP) glucuronosyltransferases; ABC - adenosine-triphosphate binding cassettes.

Irinotecan is an camptothecin analogue widely used in the treatment of gastroenteropancreatic tumors. As a prodrug irinotecan is intracellularly activated through a hydrolysis reaction catalyzed by microsomial carboxylesterase within hepatocytes. This leads to the production of the active metabolite SN-38 [133], which inhibits the topoisomerase I, a key enzyme in DNA replication which is 100 times stronger than the progenitor drug. The SN-38/topoisomerase I/DNA complex causes major breaks in the DNA replication fork, with subsequent activation of apoptosis [134] (Figure 4). The metabolism of SN-38 is mainly mediated by the cytocrome P450 enzyme CYP3A4 and CYP3A5 isoforms and by the uridine diphosphate glucuronosyltransferases (UGT), that catalyze the excretion of the drug into the bile [135, 136]. On the other hand, adenosine triphosphate binding cassettes (ABCB) allow the transport of irinotecan and its active metabolites through cell membranes, determining their distribution between cancer cells, blood and entero-hepatic circulation [137].

The most serious dose-related toxicity of irinotecan is diarrhea [138]. This effect seems to correlate with the varying degree of efficiency of UGT in conjugating SN-38 in its inactive metabolite SN-38G. In fact, in subjects with low UGT conjugating efficiency, SN-38 is directly reversed in the intestinal lumen through the bile [135]. On this basis, it is not surprising that UGT1, CYP3A4, CYP3A5, and ABCB1 polymorphisms have been explored as appealing predictors of toxicity and efficacy of irinotecan (Table 1).

● **UGT** - This superfamily of detoxifying enzymes includes two subfamilies (UGT1 and UGT2), both having endogenous and exogenous compounds as substrates. UGT1A gene is located on chromosome 2q37, and its promoter sequence (TATA box) contains a polymorphic syte, with a variable number (from 5 to 9) of dinucleotide repetitions (TA). An inverse correlation between the number of dinucleotide repetitions and the UGT1A efficiency has been demonstrated. The most common genotype in the general population is 6/6 UGT1A1^*^1, characterized by six homozygous repeat dinucleotides; the presence of 7/7 UGT1A1^*^28, typical of patients with Gilbert's syndrome, is instead characterized by a glucuronation efficiency as low as 30-50% than the normal [139]. Patients carrying the variant 7/7 who undergo irinotecan-based chemotherapy have a 4-fold increased risk of developing grade 3-4 diarrhea and neutropenia [140, 141] (Figure 4). There is lack of knowledge about the clinical significance of the other variants (5/5, 8/8 and 9/9 tandem repeats) [142].

The geographic distribution of allelic variants frequencies is heterogeneous with maximum value in African populations (23%), intermediate among Europeans (11-13%) and minimum among Asians (3%).

Based on observations from previous clinical trials, a meta-analysis of 9 studies (81 patients with lymphoma and 740 with advanced colorectal cancer) evaluated the clinical correlation between severe post-irinotecan toxicities as grade 3-4 diarrhea and neutropenia, and occurrence of UGT1A1^*^1/UGT1A1^*^28 genotypes in both homozygous and heterozygous carriers. For irinotecan dosages below 125 mg/m^2^, no differences were observed according to the genotype, whereas higher doses of the drug were associated with an increased risk of severe toxicities in subjects homozygous for the UGT1A1^*^28 variant [143] (Table 4). Consistently, another metanalysis of Caucasian patients with metastatic colo-rectal cancer who received irinotecan found a 2-to-4-time increased risk of severe neutropenia and diarrhea in UGT1A1^*^1 6/6 carriers as compared with UGT1A1^*^28 7/7 carriers. This effect was more prominent in patients treated with high doses of irinotecan, or in those receiving the drug in combination with fluorouracil [144].

**Table 4:** Genotype-phenotype correlations and recommended irinotecan dose adjustment according to UGT1A, CYP3A4^*^1B and CYP3A5^*^3 genotypes

Gene (OMIM)	nt./AA variant (rs code)	Transcriptional effects	Functional effects	Clinical effect/reported findings	Dose adjustment	Ref.
**UGT1A (#191740)**	1^*^28 A(TA)_6/7_TAA (rs34983651)	Heterozygous genotype (TA)_6/7_ 1^*^28/1^*^1	very slight reduced glucuronation efficiency than the normal	None	None	[33, 69, 140–146]
		Homozygous genotype (TA)_7/7_ 1^*^28/1^*^28	reduced glucuronation efficiency of 30-50% than the normal	(TA)_7/7_ allele increased risk of developing grade 3-4 diarrhea and severe neutropenia, especially in case of dosage >200-250 mg/m^2^. (TA)_7/7_ allele did not predict ORR to therapy.	dose reduction of 30% than the total dose (for doses >250mg/m^2^)	
CYP3A4^*^1B (#124010)	-392A>G (rs2740574)	G allele correlates with CYP3A4^*^1B higher expression	G allele increases the drug oxidative detoxification	G allele correlate with lower Irinotecan toxicities no significant clinical impact of the CYP3A4 genotype on irinotecan toxicity profile in a japanese study	None	[152, 154, 155, 158]
CYP3A5^*^3 (#605325)	6986A>G (rs776746)	G allele correlates with protein splicing defect and lower CYP3A5^*^3 expression	G allele reduces the drug oxidative detoxification	A allele correlate with lower irinotecan toxicities G allele patients with mCRC treated with IFL showed a lower response rate (RR)	None	[157, 158]
**ABCB1 (#171050)**	C3435T Ile1145Ile (rs1045642)	T allele affects mRNA stability and protein structure by reducing its function and expression	T allele causes the reduction of drug clearance by increasing toxicity risk	T allele correlates with increased risk of chemotherapy-associated toxicities and with worst ORR and OS in mCRC patients. In NSCLC patients treated with Irinotecan-Cisplatin regimen 3435T allele correlates with higher irinotecan efflux with lower AUC, higher CL and higher incidence of grade 3 diarrhea. In a French trial, in patients receiving 5 Fluorouracil, Folinic Acid more or less Irinotecan, no statistically significant correlation was found between T3435 allele and hematologic or gastrointestinal toxicities.	None	[163–165]
	C1236T Gly412Gly (rs1128503)	T allele affects mRNA stability and protein structure by reducing its function and expression	T allele causes the reduction of drug clearance by increasing toxicity risk	1236T allele correlates with prolonged exposure to irinotecan and SN-38, with a greater probability of developing ADRs but in another study this data has not been confirmed. T allele correlates with worst RR and OS in mCRC patients treated with FLIRI.	None	[137, 163, 166]

While effective in predicting toxicities, the SNPs of UGT1 appear less useful as biomarkers of efficacy. In fact, a recent metanalysis performed on 1,898 mCRC patients treated in first or second line with irinotecan showed no correlation between UGT1A1^*^28 7/7 and response rates [145].

Based on this body of evidence, in 2005 FDA recommended a 30% dose reduction for patients homozygous for UGT1A1^*^28 and candidates to irinotecan therapy at dosages >250mg/m2 [69, 146] (Table 4). No dose adjustments were indicated in case of UGT1A1^*^28 heterozygosity [146]. Similarly, the guidelines published by the Dutch Pharmacogenomics Working Group recommend dose reductions in patients with known UGT1A1^*^28 homozygous genotype [146]. In Italy, assessment of UGT1A variants is indicated in the pre-therapy setting for those patients where chemotherapy has a high risk/benefit ratio and during therapy in all cases of grade 3-4 of hematologic and/or gastrointestinal toxicities or in any case of unexpected ADRs (AIOM-SIF guidelines) [147] (Table 4).

● **CYP3A4/3A5** - The CYP3A4 and CYP3A5 genes contribute to the oxidative metabolism of irinotecan [148] (Figure 4). CYP enzymatic activity can be largely influenced by non-genetic factors as diet, ethnicity, or concomitant therapies, as well as by genetic conditions, namely the presence of polymorphic variants [149].

Fourty SNPs have been identified in the CYP3A4 gene and among them, the most studied is CYP3A4^*^1B (-392A>G, rs2740574) which is characterized by a transition from A to G in the regulatory 5’ UTR [150] (Table 1). This variant is differently distributed among various ethnic groups (high frequency among Caucasians and Afro-Americans, very low frequency among Asians) and may influence the gene transcription, leading to a substantial increase of the protein levels [151]. As result of higher CYP3A4 expression, the metabolism of irinotecan may be accelerated and the intracellular exposure to SN-38 diminished with consequent decrease of its therapeutic efficacy [152, 153]. Although limited in its power by a small sample size, a study of 30 Caucasian patients with lung or colorectal cancer showed a significant correlation between CYP3A4 genotype and irinotecan blood clearance [154]. By contrast, a pharmacokinetic analysis of 177 Japanese individuals with cancer failed to demonstrate any meaningful impact of CYP3A4 on irinotecan blood levels [155].

The polymorphism CYP3A5^*^3 (6986A>G, rs776746) is characterized by a transition from A to G in intron 3 and causes the generation of a splicing site, leading to the incorporation of an intronic sequence of 131 bp within the transcript. The ultimate consequence of this mutation is the synthesis of a truncated, non-functional protein and, indeed, carriers homozygous for CYP3A5^*^3 (G/G genotype) have very low levels of the enzyme with respect to subject with the wild type variant CYP3A5^*^1 (A/A genotype) [156] (Table 4). The clinical significance of the CYP3A5^*^3 splice variant has been investigated only in a sub-analysis of the North American Gastrointestinal Intergroup N9741 study, that investigated the combination of irinotecan, 5-fluorouracil and leucovorin in 520 patients with mCRC. Carriers of the G allele showed a response rate lower than that observed in patients bearing the A allele (29% vs 60%, p=0.0074) [157].

On the other hand, hyperfunctional CYP3A4^*^1B and CYP3A5^*^1 variants has been associated to protection from irinotecan-driven toxicities, as consequence of the accelerated drug metabolism [158] (Table 4). However, it should be noted that studies on the predictive ability of CYP polymorphisms are very difficult to interpret as result of the high inter- and intra-individual variability of CYP enzymatic activity that may primarily result from diet-, ethnicity- and therapy-related factors. At present, CYP3A4/5 genotyping does not have a definite role in customization of irinotecan-based chemotherapy [159].

**ABC transporters** - ABC transporters, among which there is P-glycoprotein (multi-drug resistance associated resistance protein 1, MDR1 or ABCB1), allow the absorption of SN-38 from plasma into hepatocytes and hence into the interstitial space. ABCB1 is a well-known drug transporter localized in the epithelial cells in the intestine, liver and kidney, contributes to the absorption of orally administered drugs, and the excretion of irinotecan and its metabolites through the bile toward the intestinal lumen and renal elimination [137]. Indeed ABC transporters are known for a long time for their ability to increase efflux of anticancer drugs from cancer cells leading to the reduction of intracellular chemotherapeutic agent levels and consequent drug insensitivity [160, 161]. ABCB1 is encoded by a gene on chromosome 7q21.12 that spans 28 exons [162]. In ABCB1 knockout mice, excretion of irinotecan and its metabolites appears to be impaired, resulting in severe alterations of the drug pharmacokinetic profile [163]. It is therefore not surprising that several variants of ABCB1 have been studied in their ability to predicit toxicity following irinotecan-based treatments (Figure 4).

The ABCB1 SNP 3435C>T (Ile1145Ile, rs1045642) consists in a silent point mutation that decreases the mRNA stability and the protein three-dimensional conformation reducing the enzyme expression [164] (Table 1). Thus, the presence of this variant determines a reduction in the excretion of irinotecan and its metabolites, causing an increased risk of chemotherapy-associated toxicities [165], (Table 4). In a Korean study of 107 patients with NSCLC who were treated with irinotecan-cisplatin, carriers of the 3435T allele underwent higher incidence of grade 3 diarrhea (*p*=0.047) [166]. However, no significant correlation between ABCB1 genotype and toxicities was found in a French, phase III, randomized trial of 5-FU and folinic acid with or without Irinotecan [167].

Similarly to 3435C>T, the ABCB1 SNP C1236T (Gly412Gly, rs1128503) is characterized by a silent mutation which alters the transcript stability and reduces protein expression [168]. In a small study of 65 patients, the 1236T allele has been associated with prolonged exposure to irinotecan and its active metabolite SN-38, and therefore higher risk of treatment-associated toxicities [137] (Table 4). In contrast, no association between increased SN-38 AUC and ABCB1 1236T was observed in another study of 85 patients with metastatic cancers treated with irinotecan monotherapy [169]. In 140 patients with mCRC treated with first-line 5-FU-irinotecan, the T allele of the C1236T, C3435T, and rs2032582 ABCB1 variants predicted poor response rates and dismal OS [165]. Given the limited available evidence, the genotyping of ABCB1 is not routinely used in clinical practice [54].

## TAXANES

**Figure 5:**
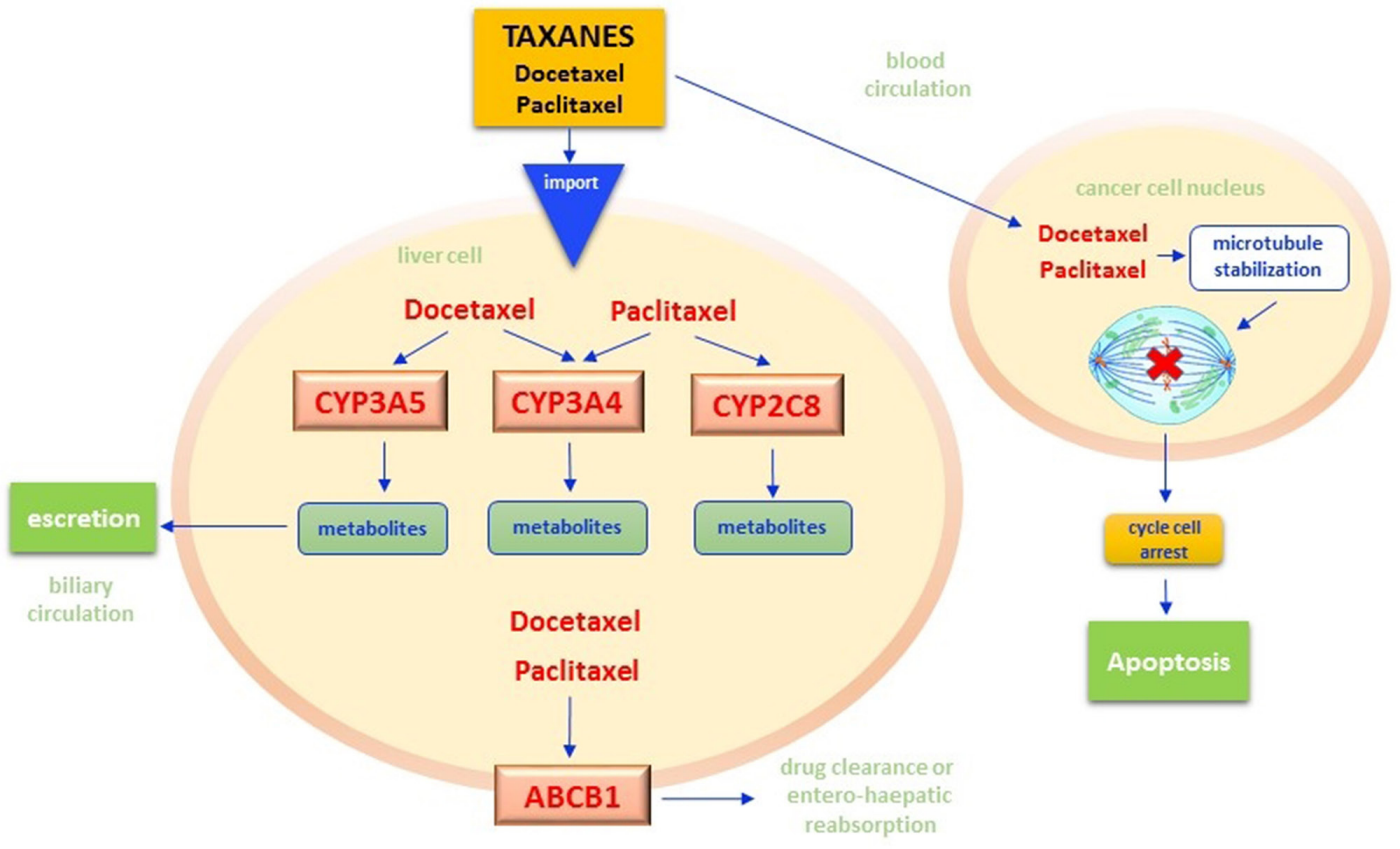
Taxanes pathway Once Paclitaxel and Docetaxel cross both plasmatic and nuclear membranes, they stabilize the nuclear beta-tubulin by inhibiting the microtubules polymerization and the mitotic melt construction. Thus, the common mechanism of action of both drugs results in the cancer cell cycle arrest in G2/M phases with the consequent apoptosis. The persistence in cytoplasm of taxane molecules and their metabolites strongly affects efficacy and toxicity of therapies. In hepatocytes, these drugs are subjected to oxidation reactions by specific isoforms of the Cytochrome P450 enzymes. In particular, CYP3A4 and CYP3A5 have docetaxel as substrate while CYP3A4 and CYP2C8 have paclitaxel. These reactions result in the synthesis of inactive metabolites that pass from the liver microsomal system into bile and then are excreted via fecal. The bioavailability of taxanes is also influenced by the functioning of ABCB1 adenosine-triphosphate binding cassettes (ABC), energy-dependent drug efflux pumps that regulate the drug clearance by influencing the balance between reabsorption from the hepatocellular system and intestinal excretion. CYP - Cytochrome P450; ABC - adenosine-triphosphate binding cassettes.

Taxanes, particularly docetaxel and its semi-synthetic derivative paclitaxel, are widely used in oncology for the treatment of lung, breast, gastric, and genital cancers [170]. Their antineoplastic mechanism is based on the inhibition of the microtubules’ assemblement with subsequent blockade of the mitotic plate formation and apoptosis [171] (Figure 5). Similarly to irinotecan, taxanes biotransformation occurs mainly within the liver, where both CYP3A4 and CYP3A5 oxidize these compounds forming inactive metabolites. ABCB1 protein, on the other hand, increase the drug clearance by balancing reabsorption from the hepatocellular system and intestinal excretion. Polymorphic variants of CYP3A4/5 and ABCB1 enzymes have been thus investigated as biomarkers of toxicity or response to taxanes [172] (Table 1).

● **ABC transporters** - Several clinical studies [173, 174] have shown a significant correlation between ABCB1 3435T allele and diarrhea of grade higher than 2 in patients with NSCLC or breast cancer receiving docetaxel. A higher frequency of mucositis was reported for carriers of ABCB1 3435T in a cohort of patients with gastric cancer undergone chemotherapy with paclitaxel and 5-FU [175]. By contrast, no association was found between ABCB1 variants and response or toxicity to paclitaxel in patients with metastatic breast cancer [176] (Table 5).

**Table 5:** ABCB1, CYP3A4^*^1B and CYP3A5^*^3 variants, potential impact on the enzyme activity and outcome of taxanes therapies

Gene (OMIM)	nt./AA variant (rs code)	Transcriptional effects	Functional effects	Clinical effect/reported findings	Dose adjustment	Ref.
**ABCB1 (#171050)**	C3435T Ile1145Ile (rs1045642)	T allele affects mRNA stability and protein structure by reducing its function and expression	T allele causes the reduction of drug clearance by increasing toxicity risk	T allele is associated with diarrhea (> grade 2) in NSCLC and breast patients and with more severe neutropenia in ovarian, breast and prostate cancer patients while C allele is associated with major risk of hematological toxicity in other studies. T allele correlates with lower PFS and higher mucositis frequency in gastric cancer patients and with dose-limiting neuropathy in breast cancer patients. In other studies conversly, 3435 C allele correlates with increased hematological toxicities.	None	[171–182]
	C1236T Gly412Gly (rs1128503)	T allele alters transcript stability by reducing protein function	T allele causes the reduction of drug clearance by increasing toxicity risk	T allele correlates with increased risk of chemotherapy-associated toxicities.	None	[137]
CYP3A4^*^1B (#124010)	-392A>G (rs2740574)	G allele correlates with CYP3A4^*^1B higher expression	G allele increases the drug oxidative detoxification	G allele correlates with decreased OS and worse clinical outcome. G allele is associated in breast cancer patients with infusion reactions but with lower risk of neuropathy	None	[151, 182, 184]
CYP3A5^*^3 (#605325)	6986A>G (rs776746)	G allele correlates with protein splicing defect and lower CYP3A5^*^3 expression	G allele reduces the drug oxidative detoxification	G allele correlates with neutropenia in breast cancer patients while in another study it is associated with lower risk of taxanes induced neuropathy	None	[179, 185, 186]

The genotype of ABCB1 has been consistently described to predict hematological toxicities in cancer patients receiving taxanes. In particular, in a study of paclitaxel-treated patients with ovarian cancer, carriers of ABCB1 3435T or 1236T experienced a severe neutropenia as compared with carriers of the C allele (*p*=0.03 and p=0.06 respectively) [177]. The association between severe neutropenia and ABCB1 3435T SNP has been subsequently confirmed in several studies of breast and prostate cancer patients treated with docetaxel [174, 178]. In one study, the ABCB1 genotype predicted not only neutropenia, but also anemia (*p*=0.044 and *p*=0.029 respectively) [179]. However, increased hematological toxicity has been demonstrated by several studies in carriers of the 3435 C allele [180, 181]. Controversial results have been reported on the correlation between ABCB1 genotype and neuropathy, the main dose-limiting toxicity of taxanes. While a study of 150 patients with early-stage breast cancer failed to find any predisposition to neurotoxicity in carriers of 1236 C>T and 3435 C>T variants [182], other analyses demonstrated a positive correlation between 3435T allele and neuropathy in breast cancer patients treated with paclitaxel and docetaxel [183, 184]. However, the presence of variant alleles is always associated with a significantly increased taxanes exposure and can be used for optimizing dosage [185].

● **CYP3A4/3A5** - Among 261 European patients with ovarian cancer treated with paclitaxel and platinum as first-line chemotherapy, the carriers of the CYP3A4^*^1B G allele were found to have inferior survival rates as compared with those showing the AA genotype, probably as result of the higher detoxifying activity of the CYP3A4^*^1B G isoform [151]. An excess of infusional reactions was also correlated to the CYP3A4^*^1B G allele variant in 70 patients with breast cancer treated with docetaxel [186] (Table 5). The SNP CYP3A5^*^3 6986G allele was revealed to predict neutropenia in patients with breast cancer receiving docetaxel [181, 187] and was associated with taxane-induced neuropathy in other studies [184, 188] (Table 5).

In 58 patients treated with taxane-based neoadjuvant chemotherapy, the A allele of CYP3A5^*^3 correlated with both favorable clinical response (Pcorr = 0.0465). Concerning the toxicity profile, the analysis of 132 patients with “gene-gene interaction” models (MDR -multifactor dimensionality reduction) analysis evaluating CYP3A5^*^3, ABCB11236C>T and ABCB1 2677G>T/A, ABCB1 3435C>T and CYP1B1^*^3 variants, showed a significant association with treatment response, but also with anemia of grade 2-4, and the dose delay/reduction induced by neutropenia (P = 0.024, P = 0.004, P = 0.026), respectively [189].

## CONCLUSIONS AND FUTURE DIRECTIONS

The germline genome dictates the expression and activity of drug receptors, downstream effectors, detoxifying enzymes, proteins and transporters within both cancer and heathy cells, and it is therefore not surprising that a plethora of studies have so far explored the role of germline genetic variants as predictors of either efficacy or toxicity of chemotherapeutics. However, despite the large body of published data, clinical implementation of SNPs of genes implicated in both pharmacodynamics and pharmacokinetics of anticancer drugs has been quite modest so far. This has been primarily caused by lack of robustness of the majority of performed studies, mostly as result of small numbers of accrued patients, enrollment of heterogeneous patient populations, as well as methodologically inconclusive study designs and lack of inter-study result replicability.

Among the most widely investigated germline genetic variants, the polymorphisms of DPYD have shown clear clinical relevance, and their genotyping is unanimously recommended for predicting the tolerance to fluoropyrimidine-based chemotherapy. However, whether DPYD analysis should be carried out in all, or only in selected patients, before or after the onset of fluoropyrimidine-related toxicities, is still a matter of debate. Future studies should compare the cost-effectiveness of different DPYD genotyping strategies in order to define the most appropriate usage setting in clinical practice, while clinical trials should formally investigate the dosages of fluoropyrimidine associated with the best efficacy/toxicity ratio in patients heterozygous for the IVS14+1G>A, 1679T>G or 2846A>T DPYD variants [190].

Clinical genotyping of UGT1A1^*^28 has been recommended for toxicity prediction in patients undergoing irinotecan-based chemotherapy, and dose adjustments have been proposed for homozygous carriers of this SNP. Whether irinotecan dose reductions may affect the efficacy of the drug is currently unknown, and future studies are needed to prospectively define the exact dosages to be used in carriers of either the wild-type or polymorphic variant of UGT1A1. In fact, there is preliminary evidence that once subjects homozygous for UGT1A1^*^28 are removed from dose-escalation studies [191], the remaining patients can tolerate doses of irinotecan higher than those currently approved for use. Given the low cost of chemotherapeutics and the high costs associated with chemotherapy-related toxicities, genotype-driven dose-finding studies of traditional chemotherapeutics should be prioritized, since they hold the potential of improving patient outcomes while reducing treatment expenses. At present the identification of germline mutations is greatly focused on cancer predisposition syndromes, where experimental and clinical evidence provided useful suggestions to introduce novel drugs, as in the case of PARP inhibitors in the treatment of patients affected by ovary cancer with inherited mutations of BRCA genes.

On the contrary, to date, there is no high-level evidence to propose that routine genotyping of germline genetic variants of other genes implicated in the metabolism of fluoropyrimidines, platinum, irinotecan, or taxanes reduces the treatment toxicities or improves chemotherapy outcomes in patients with cancer. While the level of stringent evidence demanded for formal utilization in clinical practice has not been met, SNPs of MTHFR, TYMS, ERCC1, XRCC1, GSTP1, CYP3A4/3A5 and ABCB1 might be analyzed in selected clinical scenarios, resulting particularly useful when auxiliary information are needed to inform therapeutic decisions. However, in most cases these polymorphisms are associated with a demonstrated and significantly increased or decreased chemotherapeutic exposure and can be used in pretreatment screening for optimizing dosage regimens. Furthermore, at present, the germinal characterization of a limited number of SNPs involves a simple methodological approach that is easy to use, not expensive, reliable, and highly reproducible. SNPs genotyping is performed on a whole blood sample using commercial kits based primarily on a first DNA extraction step and a subsequent analysis based on allele-specific PCR, real-time PCR or direct sequencing [192]. The interpretation of the results, which are limited to the identification of a wild type, heterozygous or homozygous variant, can be performed in most common institutions, without the requirement of additional equipment, softwares or specialized personnel. Table 6 depicts a short diagram organized as a traffic light coloring scheme: it uses the colors green for the genotypes not related to any risk, the yellow and orange colors to indicate genotypes likely related to a poorly or highly risk respectively. Finally, the red color indicates a high risk and proven by clinical observations genotype (Table 6). In this context, while no therapeutic adjustments can be recommended merely on the basis of the genotype of these genes, it is our opinion that such genomic information may be assessed among the multiple factors contributing to the final clinical decision, along with patient age, performance status, laboratory findings, and many other parameters.

**Table 6:** A “traffic light” scheme for identifying genotypes

Fluoropyrimidines					
*DPYD*	**IVS14+1G>A**	**GG**	**GA**	**AA**	
	**T1679G**	**TT**	**TG**	**GG**	Heterozygous genotype causes toxicity: administration of 50% of therapy Homozygous genotype causes sever toxicity: therapy is contraindicated
	**A2846T**	**AA**	**AT**	**TT**	
*MTHFR*	**C677T**	**CC**	**CT**	**TT**	T allele increases Fluoropyrimidines cytotoxicity
	**A1298C**	**AA**	**AC**	**CC**	C allele increases Fluoropyrimidines cytotoxicity
*TYMS*	**2R/3R repeat**	**2R/2R**	**2R/3R**	**3R/3R**	3R allele is correlated to Fluoropyrimidines resistence and low cytotoxicity
	**3R G/C**	**3R G/G**	**3R G/C**	**3R C/C**	C allele increases Fluoropyrimidines cytotoxicity
	**1494 ins/del 6b**	**Ins/Ins**	**Ins/Del**	**Del/Del**	Del allele increase toxicity and sensitivity to Fluoropyrimidine therapy
**Platinum**					
*ERCC1*	**C8092A**	**CC**	**CA**	**AA**	A allele increases Platinum genotoxicity
	**T19007C**	**TT**	**TC**	**CC**	C allele increases Platinum genotoxicity
*XRCC1*	**G28152A**	**GG**	**GA**	**AA**	A allele increases Platinum genotoxicity
*GSTP1*	**A313G**	**AA**	**AG**	**GG**	G allele increases Platinum genotoxicity
**Iirinotecan**					
UGT1A	**(TA)6/7**	**(TA)6/6**	**(TA)6/7**	**(TA)7/7**	(TA)_7/7_ genotype reduced glucuronation efficiency: reduction of 30% of therapy
*ABCB1*	**C3435T**	**CC**	**CT**	**TT**	T allele causes the reduction of drug clearance by increasing toxicity risk
*CYP3A4*	**-A392G**	**AA**	**AG**	**GG**	G allele correlate with lower Irinotecan toxicities
*CYP3A5*	**A6986G**	**AA**	**AG**	**GG**	A allele correlate with lower irinotecan toxicities
**Taxanes**					
*ABCB1*	**C3435T**	**CC**	**CT**	**TT**	T allele causes the reduction of drug clearance and increases the toxicity risk
	**C1236T**	**CC**	**CT**	**TT**	T allele causes the reduction of drug clearance and increases the toxicity risk
*CYP3A4*	**-A392G**	**AA**	**AG**	**GG**	G allele increases drug detoxification determining worse clinical outcome
*CYP3A5*	**A6986G**	**AA**	**AG**	**GG**	G allele decreases drug detoxification with lower risk of toxicities

Green areas where the risk is absent. Yellow and orange areas where the risk is poorly or highly likely respectively. Red areas where the risk is high and proven by clinical observations.

The advent of –omic sciences is rapidly transforming cancer care. New-generation sequencing technologies have propelled the recognition of molecularly distinct subclasses of tumor histotypes, allowing a more personalized preselection of treatments for cancer patients. However, by focusing on tumor genomics, -omic research has often neglected germline genomics, and while considerations on intra- and inter-tumor molecular differences are usually incorporated in therapeutic decisions, inter-individual genomic differences are limitedly considered in current clinical practice.

What is the impact of complex, multi-gene germline profiles on treatment outcomes? Can the germline profiling add clinically meaningful information beyond tumor molecular profiling? Should clinical trials be enriched of patients with similar germline genetics, rather than similar tumor genotypes? Future research is warranted to answer these questions, among others.

## Abbreviations

SNPs: Single nucleotide polymorphisms
DPYD: dihydropyrimidine dehydrogenase
UGT: uridine diphosphate glucuronosyltransferases
TYMS: fluoropyrimidines inhibit thymidylate synthetase
MTHFR: methilentetrahydrofolate reductase
NADPH/NADP+: Nicotinamide adenine dinucleotide phosphate
5-FU: 5-Fluorouracil
5’ UTR: 5’ Untranslated region
3’-UTR: 3’ Untranslated region
OS: overall survival
NER: nucleotide excision repair
MMR: mismatch repair
BER: base excision repair
ERCC1: XRCC1: excision repair cross complementation group 1; X-ray repair cross-complementing group 1
GSH: GSTP1: reduced glutathione; glutathione S-transferase protein 1
SN-38G: 7-ethyl-10-hydroxycamptothecin glucuronide
mCRC: metastatic colorectal cancer
NSCLC: non small cell lung cancer
ABCB: adenosine triphosphate binding cassettes
CYP3A4: Cytochrome P450 3A4
CYP3A5: Cytochrome P450 3A5
MDR1: multi-drug resistance associated resistance protein 1
PARP inhibitors: poly ADP ribose polymerase inhibitors
BRCA: Breast Cancer gene
